# Chronology and Sequence of Permanent Tooth Eruption in a Multi-Ethnic Urban Population

**DOI:** 10.3390/dj13080356

**Published:** 2025-08-06

**Authors:** Olivia Micheli, Maria Athanasiou, Victor Kristof, Gregory S. Antonarakis

**Affiliations:** 1 Private Practice, 1208 Geneva, Switzerland; 2 Private Practice, 5610 Wohlen, Switzerland; 3 Swiss Federal Institute of Technology, 1015 Lausanne, Switzerland; 4 Division of Orthodontics, University Clinics of Dental Medicine, University of Geneva, 1205 Geneva, Switzerland

**Keywords:** permanent teeth, tooth eruption, tooth eruption sequence, tooth eruption chronology, timing of tooth eruption

## Abstract

**Objective:** This study aimed to evaluate the mean age of eruption of permanent teeth and their clinical emergence sequence in a longitudinal sample of children from a multi-ethnic urban population. **Methods:** A total of 854 children (413 females and 441 males), aged between 4 and 13 years, were examined annually for a minimum of 4 consecutive years, as part of their annual dental screening appointment. The presence of permanent teeth was recorded at each examination. Mean and median ages, with standard deviations, of individual tooth eruption were calculated, in addition to the eruption sequence, and the analysis of the data was performed using the lognormal distribution model. Regarding the error of the method, two examiners reviewed all relevant dental screening forms, and any discrepancies were resolved through consultation with the senior author. **Results:** The sequence of permanent tooth eruption followed a consistent pattern across sexes, with distinct differences between the maxillary and mandibular arches. In the maxilla, eruption began with the first molar, while in the mandible, it started with the central incisor. Mandibular teeth generally erupted earlier than maxillary teeth, with girls experiencing earlier eruption than boys, with some exceptions, and prolonged eruption periods. No statistically significant differences were found in the timing of eruption between contralateral homologous teeth. **Conclusions:** Based on the present data, the observed sequence of tooth eruption in a multi-ethnic urban population showed similar patterns across sexes. Mandibular teeth generally erupt earlier than maxillary teeth, with girls experiencing earlier eruption than boys.

## 1. Introduction

Traditionally, it has been established that the eruption of permanent teeth occurs between the ages of 6 and 13 [1]. Reference textbooks contain average ages whereby each individual tooth type is expected to erupt [2]; however, both the age at which teeth erupt and the sequence of tooth eruption may vary from one child to another. Moreover, these reference ages often come from data originating from specific areas of the world and from many decades ago, raising the issue of possible differences due to geographical [3] and secular trends [4,5].

It is interesting to note that there are various factors that may have an impact on the timing of tooth eruption, including heredity, gender, nutrition, genetics, and socioeconomic factors [6,7]. As alluded to earlier, the timing of emergence can be affected by secular trends, which could lead to earlier eruption of the permanent teeth during the first phase of the mixed dentition and their later eruption during the second phase of the mixed dentition [4]. Girls usually present earlier eruption times than boys; hence, sex may also play an important role in the timing of tooth eruption [4,8,9,10]. Moreover, low birthweight children tend to experience a delay in the development of permanent dentition [8], while an acceleration in dental development has been observed in cases of childhood obesity [11,12]. Local and systemic conditions, genetic disorders, and genetic variants may also affect the sequence of primary and permanent tooth eruption [13,14], and it has been suggested that the type of tooth, as well as the eruption at an earlier point in the sequence of eruption, could lead to variable bilateral differences in the timing of permanent tooth eruption [15].

Geographical provenance or ethnic origin may also create variations in tooth eruption times or tooth eruption sequence. Indeed, studies have suggested that individuals of Caucasian origin have a delayed eruption time compared to other ethnic groups, and it has been shown, for example, that black populations have an earlier eruption pattern than Caucasian populations [9,10]. In general, differences in eruption times are common among different populations of the world [3], and in recent studies, a wide variability has been noticed between ethnicities, with non-Caucasians reported to have their permanent teeth erupting earlier than Europeans, Australians, or North American whites [10,16,17,18,19]. Because of the different factors influencing eruption, eruption standards for permanent teeth gain relevance when derived from the population to which they apply [20,21].

The time of eruption of permanent teeth allows the practitioner to identify an appropriate and timely treatment plan according to the patient’s dental age and future growth. Usually, the time of eruption of a tooth can be a somewhat accurate indication of early or late growth in children [22,23]. In orthodontics and paediatric dentistry, it is essential to know the age and sequence at which teeth erupt in order to plan preventive measures or to adapt treatment according to the underlying malocclusion.

As many societies are becoming more ethnically diverse and mixed, the age and sequence of tooth eruption derived from very ethnically homogeneous samples may no longer be very relevant for everyday clinical practice in a demographically shifting landscape. Thus, the aim of the present study was to evaluate the mean age of eruption of permanent teeth and their clinical emergence sequence in a longitudinal sample of children from a multi-ethnic urban population, using Geneva (Switzerland) as an example.

## 2. Materials and Methods

### 2.1. Sample

The sample for the present epidemiological study comprised children screened at the division of orthodontics of the University clinics of dental medicine of the University of Geneva (Switzerland) from 2001 to 2019, where an oral health screening of children from two large public primary schools in central Geneva was carried out. A large database of nearly 2571 children, between the ages of 4 and 13, screened over a period of almost 20 years, formed the primary sample, and anonymized data were searched. The appraisal of a larger project, which includes the current study, was carried out by the local commission for research ethics (Req-2019-00856), and it was exempt from formal approval.

The specific eligibility criteria for this study were the following: healthy children without clefting, craniofacial syndromes, or other relevant medical history; children who had not undergone or were not undergoing orthodontic treatment to eliminate the chance of teeth having been extracted; children who had not had any tooth extractions or dento-alveolar trauma, including deciduous teeth; and children who had been screened for a minimum of 4 consecutive years. The duration of follow-up varied among the children, based on their age and the frequency of their visits. Each child attended between one and nine consecutive screening appointments. However, some children had non-consecutive visits, missing a year due to illness or absence.

Children were excluded if, during the process of data extraction, inconsistencies were found or there were missing data. The initial sample was composed of 1079 children (531 girls and 548 boys), with the final sample analyzed after the exclusion of inconsistent or incomplete data, being limited to 854 children in total (413 girls and 441 boys; aged 4–13 years).

### 2.2. Data Collection

Screening forms were used for data collection. During the oral health screenings, data were gathered on variables such as sex, age, tooth presence or absence, and the number of decayed, missing, or filled teeth. A basic orthodontic assessment was also included. The screenings were carried out by dentists who were orthodontic trainees, all of whom were calibrated by a senior orthodontic consultant supervising the process. Children who continued attending the same schools were followed over time, participating in the screenings annually throughout the specified years. Data on ethnic origin were not collected as ethnic classification was not feasible due to the multi-ethnic nature of our sample, with many children having been born into families with diverse and mixed ethnic backgrounds. The complexity and richness of the population studied with regard to ethnic origin may be highlighted with a specific example of a child having been born into a family with a fusion of European, African, Asian, and South American heritage.

With regard to the presence or absence of specific permanent teeth, each tooth (with the exclusion of third molars) was classified as present or absent according to its clinical eruption. In particular, if any part of a tooth was visible, the tooth was considered clinically erupted and was recorded accordingly. If there was no visible sign of tooth eruption, the corresponding tooth was marked as absent.

### 2.3. Statistical Analysis

The selection of the best statistical model for the analysis of the data was based on the Akaike information criterion. This criterion is an estimator of the prediction error and, therefore, of the relative quality of statistical models for a given dataset. It estimates the quality of each model relative to each of the other models according to the data to be analyzed [24]. This tool was used to choose the most suitable distribution for our study, among the following ones: Weibull, lognormal, exponential, log-logistic, and generalized gamma. The lognormal distribution with the desired statistical parameters appeared to be the most relevant compared to a Weibull or Gaussian distribution. The Gaussian distribution is close to the lognormal distribution, but it accepts negative values, whereas in our research, one of the variables was age, which corresponds to positive values.

Using the lognormal distribution model, it was assumed that the eruption age of a tooth is between two screenings: first, when it was absent (denoted as 0; absence) and second, when the tooth erupted clinically (denoted as 1; presence). Therefore, the hypothesis of this study was that the tooth emerged clinically between the two recorded ages. Statistical inference allowed for the distribution of this tooth to be found using survival analysis of the probability of the tooth emerging between these two periods; thus, the data obtained in this way were more accurate. Indeed, it would bias the estimates enormously if the mean point were simply taken between the screening ages, as has been done in various research studies based on data with a Gaussian distribution model in which the mean is the median [25].

Survival analysis was used as an estimate of the time to the occurrence of tooth eruption. In survival analysis, the probability of an event occurring after a certain time is modeled, and this probability is referred to as a survival function. In our case, given our data, it was found that the survival times followed a lognormal distribution, i.e., the logarithm of the survival times followed a Gaussian-type distribution but with a positive value only, which increased its accuracy. After fitting the model to the data, the median age was reported (50% of the population), as well as the standard deviations for each tooth.

With regard to the error of the method, two examiners reviewed all relevant dental screening forms, and any discrepancies were resolved through consultation with the senior author.

## 3. Results

### 3.1. Sample

The sample of the present study was made up of 854 children, namely, 413 girls and 441 boys, aged 4 to 13 years.

### 3.2. Sequence of Tooth Eruption

**Figure 1:**
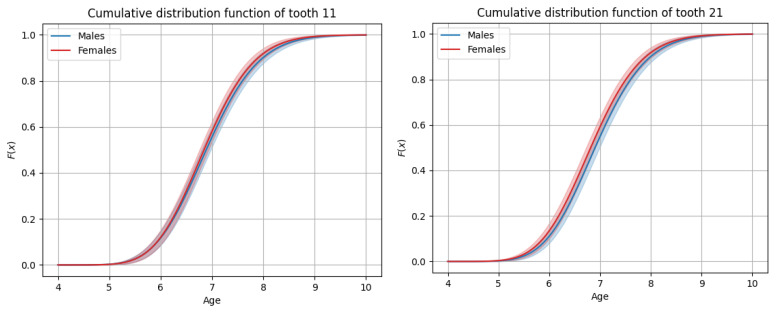

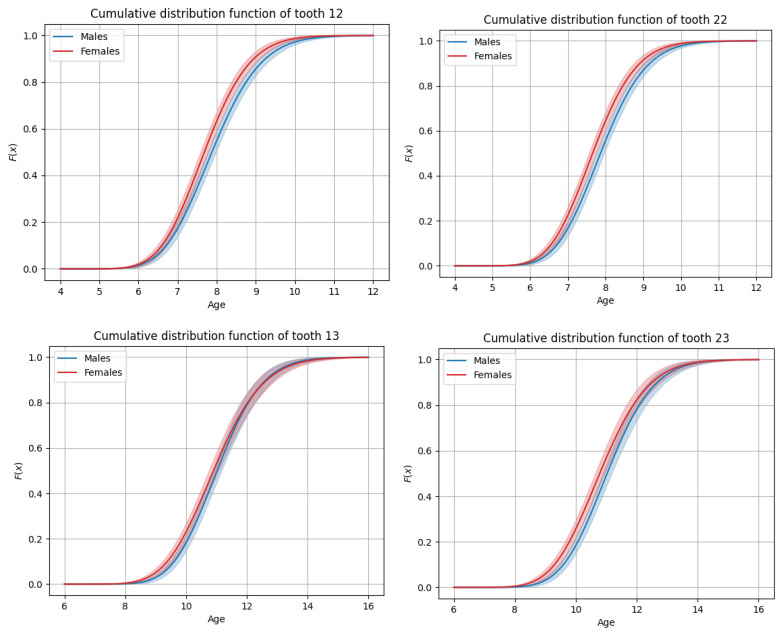
Graphs of cumulative distributions for the eruption of the maxillary anterior teeth (incisors and canines).

**Figure 2:**
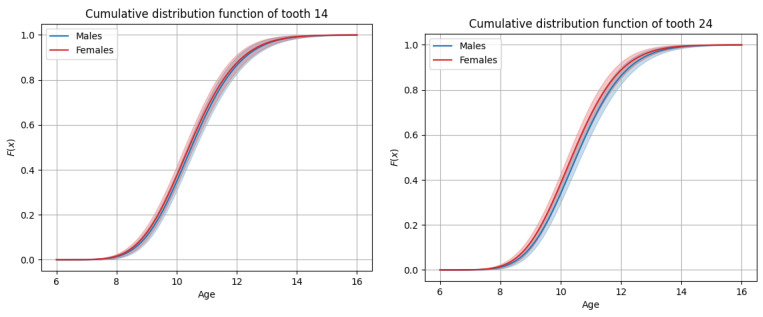

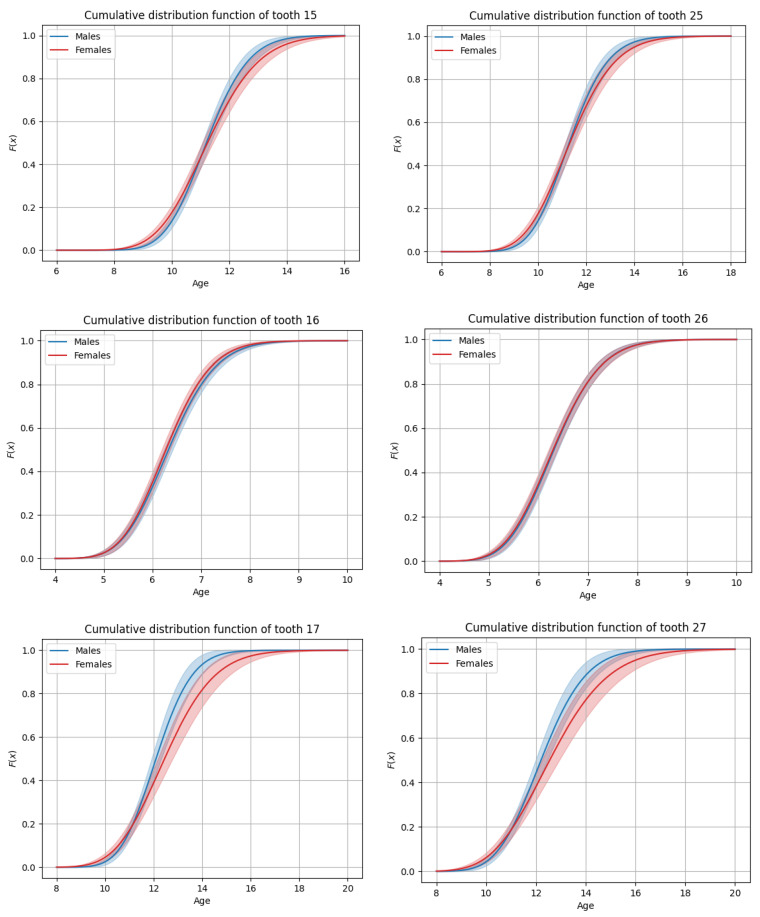
Graphs of cumulative distributions for the eruption of the maxillary posterior teeth (premolars and molars).

**Figure 3:**
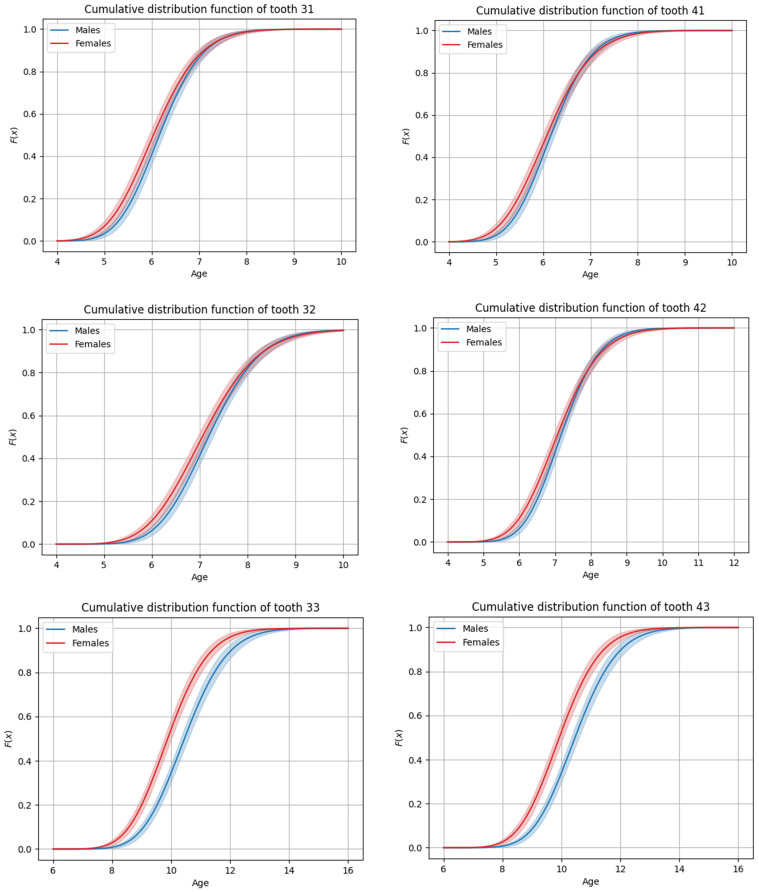
Graphs of cumulative distributions for the eruption of the mandibular anterior teeth (incisors and canines).

**Figure 4:**
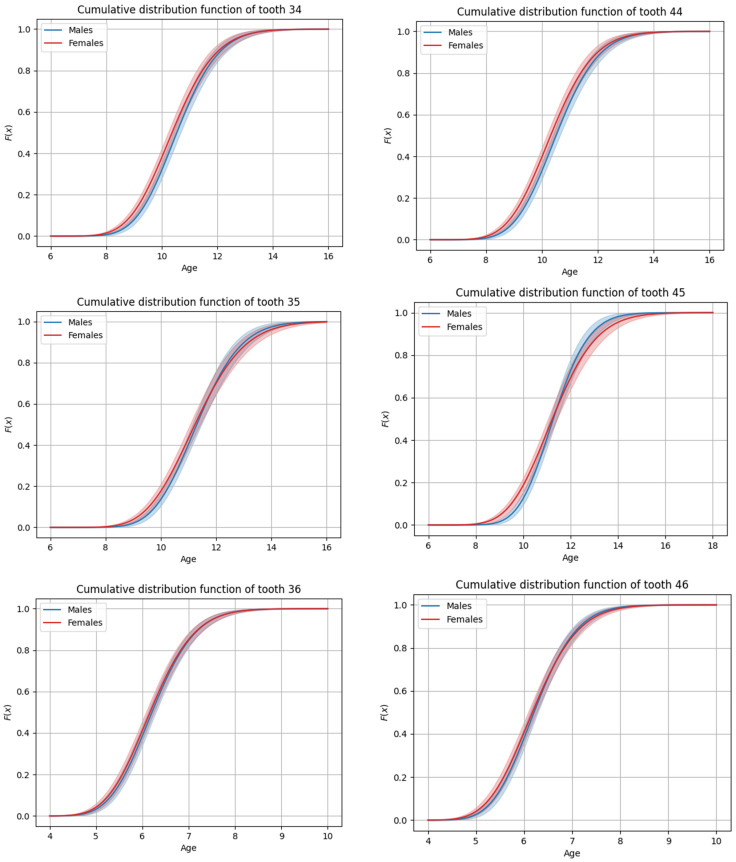

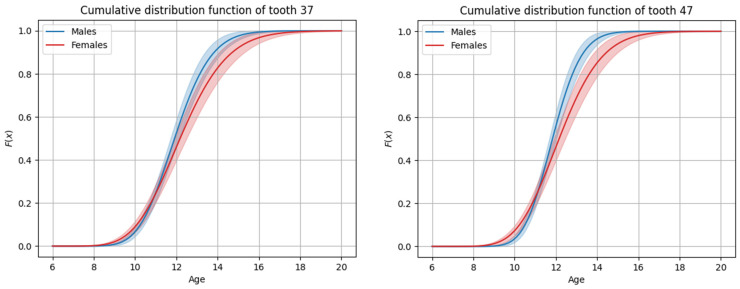
Graphs of cumulative distributions for the eruption of the mandibular posterior teeth (premolars and molars).

The sequence of permanent tooth eruption followed a consistent pattern in both boys and girls, with distinct differences observed between the maxillary and mandibular arches. In the maxilla (Figure 1 and Figure 2), the eruption sequence was as follows: first molar (M1), central incisor (CI), lateral incisor (LI), first premolar (PM1), canine (C), second premolar (PM2), and second molar (M2). In the mandible (Figure 3 and Figure 4), the eruption sequence differed slightly, with the order being: CI-M1-LI-C-PM1-PM2-M2 (Table 1 and Table 2). A notable difference in the sequence of canine and premolar eruption was observed between the arches. In the maxilla, the canine erupted after the first premolar but before the second premolar, while in the mandible, the canine erupted before both the first and second premolars. These findings illustrate distinct patterns of dental development in the upper and lower jaws. Additionally, in both boys and girls, the maxillary second molars were the last permanent teeth to erupt.

### 3.3. General Timing of Tooth Eruption

Mandibular teeth generally erupted earlier than their maxillary counterparts in both sexes. However, exceptions were noted, particularly for premolars. In boys, the maxillary PM1 and PM2 on both the right and left sides erupted earlier than their mandibular counterparts. In girls, this pattern was limited to the left maxillary PM1, which erupted earlier than the corresponding mandibular tooth. Despite these exceptions, mandibular teeth typically preceded maxillary teeth in eruption, reflecting a general trend across the sample.

### 3.4. Sex-Based Differences in Timing

Significant differences in the timing of tooth eruption were observed for certain teeth between boys and girls. Overall, the tendency found was that girls experienced earlier tooth eruption compared to boys, especially for the following teeth: maxillary right lateral incisor (0.20 years earlier; 95% CI = 0.07–0.33; *p* = 0.003), maxillary left lateral incisor (0.21 years earlier; 95% CI = 0.08–0.34; *p* = 0.001), maxillary left canine (0.23 years earlier; 95% CI = 0.06–0.40; *p* = 0.007), mandibular left central incisor (0.12 years earlier; 95% CI = 0.02–0.22; *p* = 0.020), mandibular left canine (0.57 years earlier; 95% CI = 0.42–0.72; *p* < 0.001), mandibular right canine (0.53 years earlier; 95% CI = 0.38–0.68; *p* < 0.001), and mandibular right second premolar (0.19 years earlier; 95% CI = 0.03–0.35; *p* = 0.023). The exception to this pattern was observed for the maxillary and mandibular second molars and the maxillary second premolars, which tended to erupt earlier in boys, with significant differences seen for all four second molars (0.3 to 0.4 years earlier; *p* < 0.001, except for the lower right second molar, where *p* = 0.004).

The median ages of permanent tooth eruption in girls ranged from 6.03 to 12.55 years, while in boys, eruption occurred between 6.15 and 12.20 years. Notably, the range of eruption was broader in girls, with tooth eruption starting earlier and finishing later compared to boys. This extended eruption range suggests a prolonged period of dental development in girls.

In our investigation, no statistically significant differences were found in the timing of eruption between contralateral homologous teeth in either the maxilla or mandible. Although minor variations in eruption timing were observed between right- and left-side teeth, these differences were not significant; hence, a high degree of coordination in the eruption process between the right and left sides was observed.

## 4. Discussion

In the children included in the present study, derived from a multi-ethnic urban population, the average eruption sequence in the maxilla had the following pattern: M1-CI-LI-PM1-C-PM2-M2. In the mandible, the order of eruption was as follows: CI-M1-LI-C-PM1-PM2-M2. Teeth generally erupted earlier in girls than in boys, except for the upper second premolars and the upper and lower second molars. Additionally, mandibular teeth emerged earlier than the corresponding teeth in the maxilla for both sexes, with the exception of the upper premolars, which erupted before their mandibular counterparts. Regarding the timing of emergence between contralateral homologous teeth, no statistically significant difference was found.

The classic eruption sequence for both sexes, as reported in the scientific literature, is as follows: M1-CI-LI-PM1-PM2-C-M2 for the maxilla and M1-CI-LI-C-PM1-PM2-M2 for the mandible [1,2]. This sequence has been widely used as a global reference for primary and permanent tooth eruption. However, our results showed some deviations in the eruption sequence, with the upper canine erupting before the upper second premolar and the lower central incisor before the lower first molar in both sexes.

The eruption sequence of permanent dentition has been a focal point of research interest in various countries worldwide. A continent-based comparison of our findings to those from other countries would provide valuable insights for a more comprehensive interpretation. In Europe, some researchers have reported a similar eruption pattern in the maxilla and mandible for both sexes [17,26,27]. However, in general, the eruption sequence varies by sex. In females, the sequence in the maxilla often aligns closely with our findings [28,29,30,31,32], whereas in males, different patterns are frequently observed in both jaws [29,30,31,32]. Mandibular teeth generally tend to erupt earlier than maxillary teeth, with the exception of the premolars in both sexes, and the interval of emergence is longer in females [26,29,31]. Nonetheless, in some populations, tooth emergence has been observed to last longer in males [17,27,28,32]. The upper second molars are the last teeth to erupt in the sequence for both genders [26,27,28,29,31,32], with an overall earlier eruption of all teeth generally observed in females [17,27,31,32].

In Asia, a wide variation in the eruption sequence is observed in both genders, consistent with our findings, which were primarily seen in the maxilla in girls for some populations [19,33,34,35]. In boys, there is considerable fluctuation in the sequence across both jaws [6,16,34,36,37], while in girls, certain sequences align with our results; however, the mandibular sequence differs considerably [6,16,33,34,36]. These differences in eruption patterns are mostly attributed to polymorphic variations in the eruption of C-PM2 in the maxilla and CI-M1 in the mandible. Mandibular teeth generally erupt earlier than maxillary teeth, except for the upper premolars [6,16,19,33,34,36,37,38]. However, in an Indian population, maxillary teeth were found to erupt earlier than their mandibular counterparts, suggesting earlier maturation in males compared to females [35]. Earlier tooth eruption in the sequence is generally observed in girls compared to boys [6,16,19,34,36,37,38,39], except in one study where this pattern was not demonstrated [33]. The mandibular first molar has been reported as the first tooth in the eruption sequence [6,16], while the upper second molars are the last ones [6,19,33,37]. No statistically significant differences in eruption times between the right and left sides have been observed [33,39], except in one study where bilateral differences in timing varied depending on the type of tooth, and these differences tended to be smaller for teeth erupting at an earlier point in the order of eruption and greater for those erupting at a later point [15].

In Africa, males and females exhibit the same eruption pattern in the maxilla as observed in our study, which is particularly interesting [40,41,42]. However, some variations in the mandibular sequence are noticed both in males [41,42] and in females [40,41,42]. Mandibular teeth erupt earlier than their maxillary counterparts in both sexes, except for the premolars [16,40,41], and eruption times are generally earlier in females compared to males [16,40,41,42]. The intervals of tooth emergence tend to be longer for girls compared to boys [16,40]; however, shorter intervals have also been reported [42]. The upper second molars appear to be the last teeth in the eruption sequence [40,41]. Considering studies in Australia and North America, the sequence of tooth eruption appears similar to that observed in our study, although the timing of eruption for the mandibular central incisor and mandibular first molar was reported to be exactly the same in the Australian population. Also, the mandibular teeth erupt earlier than maxillary teeth in both sexes, except for the second premolars in males. Teeth generally erupt earlier in girls than in boys, with the upper second molars emerging last in the sequence [18,43].

In general, most, but not all, findings from the aforementioned studies align well with our results. It has been reported in previous studies that, in females, permanent teeth tend to erupt later than in males, except for the premolars. This pattern is not fully consistent with our findings, since we observed that the upper second premolars and both upper and lower second molars erupt earlier in males. Additionally, mandibular teeth generally erupt earlier than their maxillary counterparts, with the exception of the premolars in some populations, a finding that agrees with our study. In our investigation, we did not find any statistically significant difference in the timing of emergence between contralateral homologous teeth, which aligns with most existing data. Furthermore, we observed that the maxillary second molars were the last teeth in the emergence sequence, an apparent and consistent finding across most studies.

Southern African children are considerably advanced in their dental development compared to the children represented in the London Atlas [44]. Black Southern African children generally show greater dental maturity compared to children of European and Asian ancestry [45], and they exhibit earlier mean ages of tooth emergence compared to populations from the USA, Europe, Australia, and Asia [40]. Additionally, Nigerian children demonstrated an earlier mean age of emergence for all permanent teeth compared to their counterparts from the USA, Belgium, Australia, and Iran. Pakistani boys showed an earlier mean age at emergence, specifically for the maxillary premolars and second molar [42], and in general, permanent teeth in Pakistani children erupt earlier than in other parts of the world, with the exception of Africa [19]. Permanent teeth tend to erupt earlier in African populations than in Asian populations [16], with the timing of emergence occurring earliest in Africa, followed by Europe, and then Asia [3], a finding that is also confirmed in our study.

In terms of the emergence pattern, Almonaitiene et al. (2012) reported that tooth emergence in the Lithuanian population closely resembles that of Finnish and German populations [29] and is in accordance with our findings of both jaws in females. Black Southern African females exhibit a similar sequence of permanent tooth emergence to females of European ancestry from Australia, Belgium, and the USA, where the upper first premolar emerges before the upper canine and the lower canine emerges before the lower first premolar. Similarly, Black Southern African males show an emergence pattern consistent with males from Australia, Belgium, and the USA, with the upper first premolar and upper canine emerging before the upper second premolar. However, in populations from Pakistan and Iran, the upper first and second premolars emerge before the upper canine. In the mandible, the sequence of tooth emergence remains consistent across these populations. It is noteworthy that Black Southern African, Nigerian, and Zambian children have longer intervals of emergence compared to their counterparts of European and Asian ancestry [40].

Concerning the emergence sequence, different patterns have been observed due to the polymorphic sequences in the maxilla and the mandible. In the maxilla, the polymorphic sequence M1-CI is noted in African and Asian populations [16]. Other common sequences include M1-CI, PM1-C, PM2-C, and PM1-PM2 in both sexes [40] and M1-CI-LI observed in 78–85% of cases in the maxilla [28], with the M1-CI-LI sequence characterized as the dominant one [30]. Additionally, the sequence C-PM2, found in 23% of men and 77% of women [46], has also been observed in both sexes in another study [18]. The M1-CI sequence is a notably common maxillary pattern, which was also found in both males and females in our study. In the mandible, we observed a polymorphism in the emergence of CI-M1 in African populations and M1-CI in Asian populations [16]. The sequences CI-M1, C-PM1, C-PM2, and PM1-PM2 have been observed in males, while females exhibit CI-M1, C-PM1, and PM1-PM2 [40]. In boys and girls, the M1-CI-LI sequence appears in 42–45% [28], while CI-M1-LI has been noted as well [30]. Furthermore, the sequence LI-M1 is more common in males, and M1-LI is more common in females [46]; in another study, the mandibular sequences CI-M1, C-PM1, and PM2-M2 were reported [18]. The polymorphic sequences M1-CI and CI-M1 in the mandible appear at varying frequencies depending on the sex and population. Some recent data even suggest that the C-PM2 sequence in the maxilla and the PM1-PM2 sequence in the mandible are associated with more dental crowding [47].

Knowing the timing and emergence sequence of permanent teeth can assist practitioners in making accurate diagnoses and proposing treatments that are tailored to the patient’s dental age and potential for future growth. Our findings are based on a specific multi-ethnic urban local population, and as such, they cannot serve as a reference for other populations around the world, but they provide valuable insights specific to this cohort. Interestingly, they align closely with those from studies conducted in European populations, underscoring that the primary intercontinental differences relate to the eruption sequence and timing of tooth emergence. Consequently, considering these population-specific variations could enhance accuracy in assessments across diverse groups. Vandana et al. (2024), in their systematic review and meta-analysis, confirmed that teeth generally erupt earlier in females than in males and earlier in the mandible than in the maxilla [3]. This is confirmed in more recent studies, which, again, found that tooth eruption occurs earlier in females and in the mandible; they also found earlier tooth eruption in those with a normal body mass index and high socioeconomic status [48]. Other studies, however, did not find such a clear association between tooth eruption and body mass index or weight [49,50]. The widely referenced “Chronology of the Human Dentition” by Logan and Kronfeld, established in 1933, has traditionally served as a global standard for both primary and permanent tooth eruption. However, the development of transcontinental tooth eruption chronology charts may be beneficial in accounting for social, environmental, and genetic variations across populations.

## 5. Conclusions

Based on this study and within its limitations, the observed sequence of tooth eruption in this multi-ethnic urban population of Geneva (Switzerland) showed similar patterns across sexes. Mandibular teeth generally erupted earlier than maxillary teeth, with some exceptions. Girls generally experienced earlier eruption than boys and prolonged periods of eruption. It could be stated that transcontinental eruption charts could provide a comprehensive understanding of differences in eruption patterns across populations, allowing for more accurate assessments and interventions.

## Figures and Tables

**Table 1:** Age of emergence and eruption sequence of permanent teeth in males.

Maxilla				Mandible			
Tooth	Median age	Average age	Standard deviation	Tooth	Median age	Average age	Standard deviation
11	6.89	6.93	0.81	31	6.16	6.20	0.72
12	7.88	7.94	1.00	32	7.18	7.23	0.85
13	11.02	11.08	1.18	33	10.45	10.52	1.16
14	10.49	10.57	1.30	34	10.54	10.61	1.19
15	11.20	11.26	1.16	35	11.30	11.37	1.27
16	6.33	6.37	0.77	36	6.21	6.25	0.74
17	12.10	12.16	1.18	37	11.91	12.00	1.40
21	6.90	6.95	0.80	41	6.15	6.19	0.69
22	7.87	7.92	0.95	42	7.17	7.22	0.84
23	11.03	11.09	1.21	43	10.46	10.52	1.15
24	10.52	10.59	1.29	44	10.51	10.58	1.20
25	11.28	11.35	1.28	45	11.27	11.33	1.18
26	6.31	6.35	0.76	46	6.21	6.25	0.70
27	12.20	12.29	1.43	47	11.84	11.89	1.11
**Maxillary eruption sequence:** **M1-CI-LI-PM1-C-PM2-M2**				**Mandibular eruption sequence:** **CI-M1-LI-C-PM1-PM2-M2**			

**Table 2:** Age of emergence and eruption sequence of permanent teeth in females.

Maxilla				Mandible			
Tooth	Median age	Average age	Standard deviation	Tooth	Median age	Average age	Standard deviation
11	6.84	6.89	0.77	31	6.03	6.08	0.78
12	7.68	7.74	0.92	32	7.05	7.11	0.98
13	10.90	10.98	1.29	33	9.89	9.95	1.11
14	10.41	10.49	1.30	34	10.37	10.45	1.25
15	11.24	11.33	1.42	35	11.23	11.32	1.42
16	6.28	6.32	0.74	36	6.17	6.21	0.76
17	12.45	12.56	1.63	37	12.18	12.32	1.82
21	6.81	6.86	0.79	41	6.07	6.12	0.78
22	7.66	7.71	0.91	42	7.06	7.13	0.95
23	10.78	10.86	1.26	43	9.93	9.99	1.11
24	10.36	10.44	1.27	44	10.31	10.39	1.23
25	11.31	11.40	1.50	45	11.23	11.33	1.48
26	6.29	6.34	0.77	46	6.17	6.22	0.75
27	12.55	12.69	1.88	47	12.16	12.27	1.65
**Maxillary eruption sequence:** **M1-CI-LI-PM1-C-PM2-M2**				**Mandibular eruption sequence:** **CI-M1-LI-C-PM1-PM2-M2**			

## Data Availability

The data that support the findings of this study are available from the corresponding author upon reasonable request.
